# Practical Hints

**Published:** 1877-01-20

**Authors:** 


### PRACTICAL HINTS.

Dr. F. L. Gerald, of Massachusetts, in *Eclectic Medical Journal*, publishes a paper on Uterine Pathology, from which we cull the following suggesticns and formula, which, though partaking in some measure both of homeopathy and of electicism, so-called, seem to possess practical importance.

Upon the subject of the various sympathetic and reflex disorders resulting from uterine disease, he remarks if the the appetite is poor with distressed feeling after meals, use the following:

R. Tinct. nux vomica, gtt. xxx
" Collinsonia canadinsis.............................edr. iij
Water ...............................oz. iv
M. S. A teaspoonful before each meal, giving a teaspoonful of the elixir of bismuth after each meal.

Well adapted to many forms of indigestion.

If the bowels are constipated, the tongue having a yellow coating at its base, use the following

LAXATIVE PILL.

R. Euonymin,
Laptandrin......... aa...........gr. xx
Podophylin.......................gr. vi
Pulv. capsicum...................gr. vij
M. Ft. Pills No. xx.

Dose one to three twice or three times a week.

If the system is in a state of anænmia and nervous prostration, nse the following

NERVE TONIC AND ALTERATIVE.

R. Syrup prun. virg. or glycrine oz. iv
Tinct. ferri. chlo................dr. iij
Acid phosphoric dilut..........dr. iv
M. Dose—A teaspoonful in a little cold water after each meal.

FOR STRUMOUS CASES,

R. Tinct. phytolacca ...............dr. iv
Iodid. ammonium ...............dr. ij
Syr. prun. virg.....................oz. iv
Spts. gaultheria ...................dr. ss
M. Dose—A teaspoonful three times per day.

When a patient complains of a bad feeling in the head, with nervousness and restlessness, and is in fear of impending danger, use the following

NERVINE.

R. Tinc. cactus grand..............dr. iss
Tinc. pulsatilla.... ...............dr. i
Water..............................oz. iv
M. Dose—A teaspoonful every four hours.

For a feeling of weight and dragging in the uterine region:

R. Fluid ext. helonias dioica.....dr. ij
Fluid ext. mitchella repens...dr. iv
Fluid ext. canlophylum, thal.dr. iss
Spts. gaultheria..................dr. ss
Syr. prun. virg...................oz. iv.
M. Dose—A teaspoonful three or four times a day.

In

IRRITABLE BLADDER AND PAINFUL MICTURATION.

R. Tinc. stramonium................dr. i
Bi carb soda.........................dr. i
Water.................................oz. iv
M. Dose—teaspoonful every three hours.

